# Graves' disease presenting as pseudotumor cerebri: a case report

**DOI:** 10.1186/1752-1947-5-68

**Published:** 2011-02-15

**Authors:** Ester Coutinho, Ana M Silva, Cláudia Freitas, Ernestina Santos

**Affiliations:** 1 Serviço de Neurologia, Hospital Santo António, Largo Professor Abel Salazar, 4099-001 Porto, Portugal; 2 Serviço de Endocrinologia, Hospital Santo António, Largo Professor Abel Salazar, 4099-001 Porto, Portugal

## Abstract

**Introduction:**

Pseudotumor cerebri is an entity characterized by elevated intracranial pressure with normal cerebrospinal fluid and no structural abnormalities detected on brain MRI scans. Common secondary causes include endocrine pathologies. Hyperthyroidism is very rarely associated and only three case reports have been published so far.

**Case presentation:**

We report the case of a 31-year-old Luso-African woman with clinical symptoms and laboratory confirmation of Graves' disease that presented as pseudotumor cerebri.

**Conclusion:**

This is a rare form of presentation of Graves' disease and a rare cause of pseudotumor cerebri. It should be remembered that hyperthyroidism is a potential cause of pseudotumor cerebri.

## Introduction

Pseudotumor cerebri (PTC) is an entity characterized by elevated intracranial pressure with normal cerebrospinal fluid (CSF) and no structural abnormalities detected on brain MRI scans. The neurological symptoms and signs can be totally attributed to intracranial hypertension, and these include headaches, transient visual obscurations, visual loss and intracranial tinnitus, papilledema being the hallmark of PTC. This syndrome includes both idiopathic and secondary causes. Common secondary causes include endocrine pathologies. Thyroid disturbances have a unique correlation, since hypothyroidism, hyperthyroidism and thyreostimulin suppression hormone therapy have all been reported in association with this disorder. Hyperthyroidism is very rarely associated with the disorder and only three case reports [1-3] have been published to date, one of them [3] in association with hypovitaminosis A.

## Case presentation

**Figure 1:**
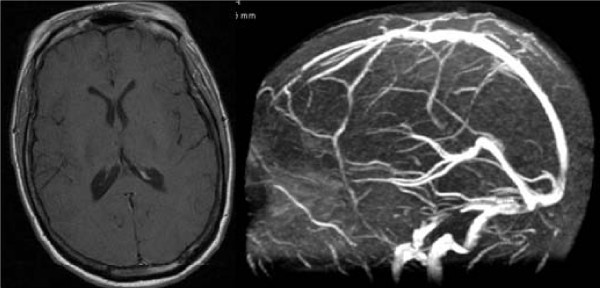
**Brain MRI scan with venography**. A brain MRI scan showing normal morphology, ventricular dimensions and venous drainage.

**Figure 2:**
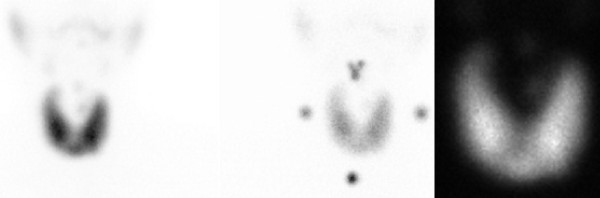
**Thyroid scintigraphy**. Technetium 99 m pertechnetate thyroid images showing the goiter with diffusely increased radiotracer uptake.

A 31-year-old Luso-African woman who was a law student was admitted to our department. She was slim (body mass index (BMI) of 22), with no relevant medical history and not taking any drugs. She presented with progressive symptoms: persistent headache and vomiting that had lasted for six months accompanied by visual disturbance ('blurred vision') in the last few days. She also stated she had pain with ocular movements and neck stiffness. She had also shown clinical symptoms and signs (tremor, diarrhea, tachycardia, heat intolerance and exophthalmus) for the past five months suggestive of hyperthyroidism. On neurological examination she presented with bilateral papilledema and nuchal rigidity. Goldmann visual fields showed bilateral enlargement of the blind spot. Results of a brain MRI with venography were normal (Figure 1). Her CSF opening pressure was raised (410 mm of water), had no cells, with normal glucose and protein levels. A lumbar puncture to relieve pressure was not performed; the diagnostic lumbar puncture caused no symptomatic relief. Blood analysis showed a totally suppressed thyroid-stimulating hormone level of 0.01 mIU/L (normal 0.35 to 4.50 mIU/L) and a free T3 level of 31.6 pmol/L (normal 3.5 to 6.5 pmol/L). A thyroid-stimulating hormone receptor antibody value of 49 U/L and thyroid scintigraphy showing a diffusely increased radiotracer uptake goiter (Figure 2) confirmed the diagnosis of Graves' disease. Other etiological causes were excluded via blood and CSF analysis, namely infectious and immunological diseases. Pharmacological causes were excluded since she was not taking any drugs. Treatment with tiamazol (30 mg/day), propranolol (120 mg/day) and acetazolamide (1500 mg/day) was given. After 10 days of treatment she had no headaches, nausea or vomiting. The papilledema resolved in the following months. She continued acetazolamide treatment for four months and is currently being treated with tiamazol. Her thyroid function slowly recovered.

## Conclusion

Considering that other causes were excluded and there was neurological improvement once hyperthyroidism treatment was started, a relationship between hyperthyroidism and PTC can be assumed. Although acetazolamide was also used during the symptomatic phase, and this could represent a confounder, we are convinced that the thyroid disease treatment was the major reason for improvement. This is a rare form of presentation of Graves' disease and a rare cause of PTC.

The pathophysiologic basis of PTC is still a matter of debate, but a relationship has been established [4,5] with elevated intracranial venous pressure. The increase in resistance of CSF absorption is thought to be caused by an insufficiently high driving pressure gradient from the subarachnoidal space to the venous system. Thyroxine, being a major regulator of sodium transport, can contribute to altered CSF dynamics. The effect of thyroid hormone raising venous pressure may justify the association between those two entities. In fact, there is a previously reported association between thyrotoxicosis and cerebral vein thrombosis [6], with additional procoagulant influences probably required in such cases.

We would like to emphasize that hyperthyroidism should be considered among the causes of PTC and that this association should be given further attention. Optic fundus examination with screening for papilledema in patients with thyroid diseases could detect more patients with intracranial hypertension, helping to prevent visual sequelae.

## Consent

Written informed consent was obtained from the patient for publication of this case report and any accompanying images. A copy of the written consent is available for review by the Editor-in-Chief of this journal.

## Competing interests

The authors declare that they have no competing interests.

## Authors' contributions

EC analyzed and interpreted the data regarding the neurological presentation of the disease, and was the major contributor in writing the manuscript. AMS analyzed and interpreted the data regarding the endocrinological aspects of the case. CF analyzed the endocrinological data and is responsible for the follow-up of our patient. ES analyzed the neurological data, is responsible for the follow-up of our patient and was an active contributor for the writing of the manuscript. All authors read and approved the final manuscript.

## References

[B1] Dickman MS Somasundaram M Brzozzwski L Pseudotumor cerebri and hyperthyroidism N Y State J Med 1980 80 1118 1120 6930579

[B2] Merkenschlager A Ehrt O Müller-Felber W Schmidt H Bernhard MK Reversible benign intracranial hypertension in a child with hyperthyroidism J Pediatr Endocrinol Metab 2008 21 1099 1101 19189707 10.1515/jpem.2008.21.11.1099

[B3] Roos RA Van der Blij JF Pseudotumor cerebri associated with hypovitaminosi A and hyperthyroidism Dev Med Child Neurol 1985 27 246 248 10.1111/j.1469-8749.1985.tb03776.x 3838956

[B4] Skau M Brennum J Gjerris F Jensen R What is new about idiopathic intracranial hypertension? An updated review of mechanism and treatment Cephalalgia 2005 26 384 399 10.1111/j.1468-2982.2005.01055.x 16556239

[B5] Bateman GA Arterial inflow and venous outflow in idiopathic intracranial hypertension associated with venous outflow stenoses J Clin Neurosci 2008 5 402 408 10.1016/j.jocn.2007.03.018 18242091

[B6] Squizzato A Gerdes VEA Brandjes DPM Büller HR Stam J Thyroid diseases and cerebrovascular disease Stroke 2005 36 2302 2310 10.1161/01.STR.0000181772.78492.07 16179578

